# Arsenic Toxicity-Induced Physiological and Metabolic Changes in the Shoots of *Pteris cretica* and *Spinacia oleracea*

**DOI:** 10.3390/plants10102009

**Published:** 2021-09-25

**Authors:** Veronika Zemanová, Daniela Pavlíková, František Hnilička, Milan Pavlík

**Affiliations:** 1 Department of Agro-Environmental Chemistry and Plant Nutrition, Faculty of Agrobiology, Food and Natural Resources, Czech University of Life Sciences Prague, Kamýcká 129, 165 00 Prague 6, Czech Republic; pavlikm@af.czu.cz; 2 Department of Botany and Plant Physiology, Faculty of Agrobiology, Food and Natural Resources, Czech University of Life Sciences Prague, Kamýcká 129, 165 00 Prague 6, Czech Republic; hnilicka@af.czu.cz

**Keywords:** amaranthaceae family, arsenic contamination, fern, metalloid, pteridaceae family, spinach, abiotic stress

## Abstract

Arsenic is a ubiquitous toxic element that can be accumulated into plant parts. The present study investigated the response of *Pteris cretica* and *Spinacia oleracea* to As treatment through the analysis of selected physiological and metabolic parameters. Plants were grown in pots in As(V) spiked soil (20 and 100 mg/kg). Plants’ physiological condition was estimated through the determination of elements, gas-exchange parameters, chlorophyll fluorescence, water potential, photosynthetic pigments, and free amino acid content. The results confirmed differing As accumulation in plants, as well as in shoots and roots, which indicated that *P. cretica* is an As-hyperaccumulator and that *S. oleracea* is an As-root excluder. Variations in physiological and metabolic parameters were observed among As treatments. Overall, the results revealed a significant effect of 100 mg/kg As treatment on the analysed parameters. In both plants, this treatment affected growth, N, Mg, S, Mn, and Zn content, as well as net photosynthetic rate, chlorophyll fluorescence, and total free amino acid content. In conclusion, the results reflect the similarity between *P. cretica* and *S. oleracea* in some aspects of plants’ response to As treatment, while physiological and metabolic parameter changes related to As treatments indicate the higher sensitivity of *S. oleracea*.

## 1. Introduction

Arsenic is a poisonous metalloid with potent carcinogenic and mutagenic properties [1], presenting a high ecotoxicological risk. In the soil, As content increases through various anthropogenic activities (e.g., mining) or naturally through geochemical processes [2]. In soil, water, and air, As exists in many chemical forms with variable degrees of mobility, bioavailability, and toxicity to plants [3]. Factors affecting these parameters include the As concentration in the soil, As species, the type of plant species, and other soil properties controlling As accessibility, accumulation, and fate in soils, microorganisms, and plants [4]. Overall, inorganic As is more lethal and movable than the organic form [2]. The most abundant As form is As(V) under oxidising conditions, while As(III) predominates under reducing conditions, and these two forms are inter-convertible [5]. Inorganic As(V) remains mostly in the free form as an anion species, whereas As(III) can be bound to oligopeptide phytochelatins or proteins. The As(III) could serve as a storage deposit, whereas free forms could be excreted [6]. Although much progress has been made in understanding the mechanism of As uptake, translocation, and accumulation, several knowledge gaps still exist [7].

Plants’ intake of As can hardly be downregulated, as it is often mediated by essential element transporters [8]. Arsenic exposure has an adverse effect on the morphological (e.g., chlorosis), physiological (e.g., growth processes inhibition), and biochemical (e.g., oxidative stress) responses of plants [9,10]. Inside plant cells, both As(V) and As(III), including their conversion, induce oxidative stress by enhancing the production of reactive oxygen species, which affects the regulation of a diverse range of metabolic pathways [1,11]. The induction of oxidative stress is the main process underlying As toxicity in plants [12]. Furthermore, As acts by impairing mitochondrial enzymes, thereby causing a halt in cellular respiration and uncoupling oxidative phosphorylation [6]. Arsenate does not react directly with the active sites of enzymes [6], but this form strongly interacts with sulfhydryl groups in proteins, interfering with cellular functions [5,13].

All plant tissues are prone to being adversely affected by As [12]. However, leaves are key interfaces between plants and their surrounding environment and are important to photosynthesis [14]. In the context of As stress, the level of photosynthetic apparatus damage is chiefly related to the dosage of As treatment and application form [11]. Furthermore, leaf structural properties, such as mesophyll thickness, mesophyll surface area, and leaf reflectance, are adversely affected by As [15].

Carbon, N, and S uptake and metabolism are impacted by As exposure [16,17]. Studies have shown that As affects the amino acid (AA) content in tissues of different plant species [11,18,19,20,21]. The AAs are necessary for metabolic processes and the transport and storage of all nutrients, such as carbohydrates, proteins, vitamins, elements, water, and fats [22]. Some AAs are involved in N fixation, and some free AAs have been shown to have a protective chelating role in metal/metalloid stress tolerance [2]. Furthermore, several AAs play key roles as precursors for secondary metabolite biosynthesis [22].

Plants exhibit a wide variability in their response to As [20]. The ability of plants to survive in the presence of an element that is otherwise toxic is defined as tolerance [5]. Tolerant species can be classified as non-As-hyperaccumulators and As-hyperaccumulators [20]. The latter have high tolerance to As and are able to concentrate > 1000 mg As/kg dry weight in shoots compared to non-As-hyperaccumulators [23]. In As-hyperaccumulating plants, the main physiological processes, such as photosynthesis, respiration, and water and nutrient metabolism, should not be disturbed by high As accumulation [24]. Many plants have been reported to tolerate As in contaminated soils but not as As-hyperaccumulators because they accumulate As very slowly over an extended period of time and sequestered As predominantly in the roots [10,25].

To avoid As toxicity, tolerant plants utilise various strategies, such as reducing the concentration of toxic elements at sensitive sites inside a plant cell [5]. Once inside the plant cell, most As(V) is reduced to As(III), as only the trivalent form can undergo detoxification [8]. The cellular detoxification of As(III) in non-As-hyperaccumulators involves As complexation with phytochelatins respectively nonprotein thiols and storage of these complexes in vacuoles [26,27], and can be characterized as adaptive resistance. Compared to non-As-hyperaccumulators, As-hyperaccumulators had less of a tendency for complex formation [13,26,27]. It has been shown that in tolerant ferns, most As(V) is reduced by ACR2 reductase to As(III), which is subsequently transported by ACR3 transporter to the leaves [28].

Overall, vegetables are generally highly sensitive to metal/metalloid stress [29], and leafy vegetables have shown higher accumulation of metals/metalloids, including As [30]. *Spinacia oleracea* is one of the most valuable leafy vegetables, possessing large surface areas, relatively high growth rates, high nutrient content, and elevated metal/metalloid absorption rates [31]. It is widely cultivated and studied worldwide for the accumulation of metals/metalloids [32]. Nevertheless, there is a lack of information on comparing differences in the response of *S. oleracea* versus As-hyperaccumulators to As stress.

This study aimed to investigate responses to As treatment in two plant species differing in As accumulation and tolerance. It was expected that *Pteris cretica* ‘Albo-lineata’ would have a different response to As toxicity than *Spinacia oleracea* ‘Monores’. For this purpose, the changes in physiological and metabolic parameters in the shoots of both plant species were studied under two different As treatments in a pot experiment. Arsenic bioaccumulation and translocation in each plant species was determined, and in relation to these abilities, the effect of As on the nutrient content, leaf gas-exchange parameters, chlorophyll fluorescence, water potential, and photosynthetic pigments, as well as the regulation of free AAs, were evaluated.

## 2. Results

### 2.1. Arsenic Accumulation and Translocation

**Figure 1:**
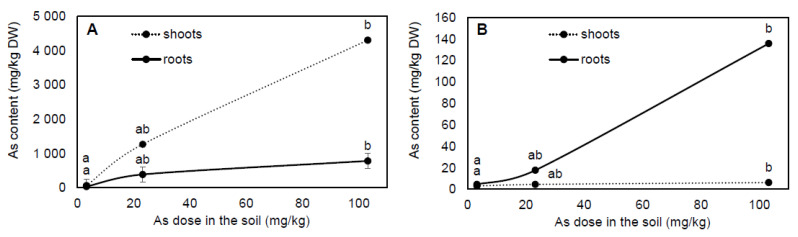
Effect of As treatments on shoot and root dry biomass of *Pteris cretica* (**A**) and *Spinacia oleracea* (**B**) in the pot experiment. Values represent the mean ± SE of three biological and two technical replicates per sample. Data with the same letter are not significantly different. Different letters indicate significant differences (*p* ≤ 0.05) among treatments according to the Kruskal-Wallis test. The background soil pseudo-total As content was 16 mg As/kg soil. The difference between control and individual As treatments was the spiked As dose plus the 20% As extraction efficiency.

The difference in plants’ response to As treatment was shown in As content (Figure 1A,B), which was significantly different between shoots and roots of plants, as well as between plant species. However, As accumulation was treatment-dependent for both plant species (r = 0.95–0.99, *p* ≤ 0.001). Arsenic content in *P. cretica* reached from 95.4 to 5088.5 mg/kg DW with the highest As ratio in the shoots (64.4–84.6%; Figure 1A). The ratio of As in the roots was 15.4–35.6%. In *P. cretica*, the results of BF and TF, both >1, showed very high As bioaccumulation and high translocation in the biomass, confirming *P. cretica* as an As-hyperaccumulator (Table 1). Compared to *P. cretica*, the As content in *S. oleracea* was 41-fold lower on average and reached from 7.6 to 141.9 mg/kg DW with an As accumulation ratio of 4.4–40.1% in the shoots and 59.9–95.6% in the roots (Figure 1B). In *S. oleracea*, values of BF were <1, except roots of As100 treatment, which indicated that *S. oleracea* was able bioaccumulated low As content. Similar to BF, TF values were <1 in all treatments and indicated very low As translocation from roots to the shoots of this plant species (Table 1). The trend of As bioaccumulation in the roots suggested that *S. oleracea* is a root excluder (Figure 1B). The higher As treatment (As100) had a contrasting response in both plant species based on the TF and BF of shoots, which were significantly increased by As100 treatment in *P. cretica* and significantly decreased in *S. oleracea*.

### 2.2. Effect of Arsenic on Plant Growth

**Figure 2:**
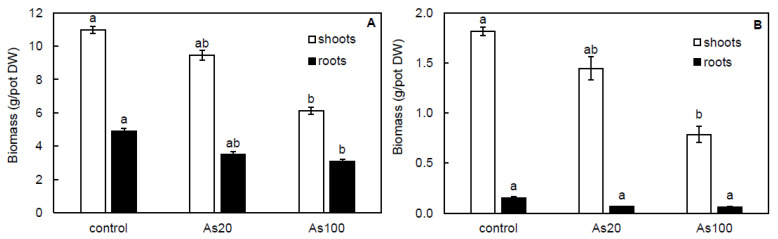
Effect of As treatments on shoot and root dry biomass of *Pteris cretica* (**A**) and *Spinacia oleracea* (**B**) in a pot experiment. Values represent the mean ± SE of three biological replicates per sample. Data with the same letter are not significantly different. Different letters indicate significant differences (*p* ≤ 0.05) among treatments according to the Kruskal-Wallis test. Treatment abbreviations: control—0 mg As/kg soil; As20—20 mg As/kg soil; As100—100 mg As/kg soil.

Arsenic treatments affected the shoot and root dry biomass of *P. cretica* (r = –0.98 and –0.76, respectively; *p* ≤ 0.01); however, a significant decrease was confirmed only for the As100 treatment (Figure 2A). Compared to the control, As100 treatment decreased shoots and roots of *P. cretica* by 44 and 37%, respectively. Similar to *P. cretica*, the shoot dry biomass of *S. oleracea* was affected by As treatment (r = –0.95, *p* ≤ 0.01), and compared to the control, As100 treatment significantly decreased the shoot dry biomass by 57% (Figure 2B).

### 2.3. Effect of Arsenic on Nutrient Content

A clear difference in response to As treatments between plants was shown by the nutrient content in shoots (Table 2), which were significantly different between plant species, except Fe at As100 treatments and Ni in the control and As20 treatment. In both plant species, a treatment-dependent trend for some nutrients was observed (Table S1). In both plant species, Ni content was not significantly affected by As treatment (Table 2).

Nitrogen content in *P. cretica* was on average 1.5-fold lower compared to *S. oleracea* (Table 2). In *P. cretica* and *S. oleracea* shoots, the As100 treatment increased N content by 16 and 11%, respectively. Similar results were observed for S content in the shoots of both plant species. Shoots of *P. cretica* and *S. oleracea* treated with As20 and As100 accumulated a higher S content compared to the control; however, the increase of 83% (*P. cretica*) and 51% (*S. oleracea*) was significant only for As100 treatment (Table 2).

The contrasting effect of As treatment in *P. cretica* and *S. oleracea* shoots was observed for Mg, Mn, Zn, and Fe. *Pteris cretica* shoots treated with As20 and As100 accumulated higher Mg content compared to the control (by 12 and 24%, respectively); however, the increase was only significant for As100 treatment (Table 2). A contrasting response to As20 and As100 treatments was observed in *S. oleracea* shoots, which accumulated a lower Mg content compared to the control (by 7 and 24.5%, respectively); however, the decrease was only significant for As100 treatment (Table 2). Similarly, As100 treatment increased Mn and Zn content in *P. cretica* shoots by 16 and 38%, respectively, but decreased by 26.5 and 45%, respectively, in *S. oleracea* shoots (Table 2). In *P. cretica* and *S. oleracea* shoots, As20 and As100 treatments showed similar trends in Fe content as that observed for Mg content; however, a significant effect was determined only for As20 treatment (Table 2). Compared to the control, As20 treatment increased the Fe content (91%) in *P. cretica* shoots, while in *S. oleracea* shoots, Fe content was decreased (34%).

Phosphorus and Cu showed different effects of As treatment in *P. cretica* and *S. oleracea* shoots (Table 2). Phosphorus content was increased by As20 treatment (40%) in *P. cretica* shoots, while it was decreased by As100 treatment (33%) in *S. oleracea* shoots. Similarly, Cu content was increased by As20 treatment (36%) in *P. cretica* shoots, but decreased by As100 treatment (13.5%) in *S. oleracea* shoots.

### 2.4. Effect of Arsenic on Photosynthetic Pigments

The increased As treatment had a negative impact on the total content of chlorophylls (Chl_tot_) and carotenoids (Car) in *P. cretica* and *S. oleracea* (Table 3); however, the content of Chl_tot_ and Car was treatment-dependent only in *S. oleracea* shoots (r = −0.72 and −0.76, respectively, *p* ≤ 0.05). Photosynthetic pigment content was not significantly different between plants. Although, in *P. cretica*, As treatment affected Chl_tot_ and Car by 36 and 40%, on average, respectively, the effect of As treatment was not significant (Table 3). Similar to *P. cretica*, the Car content in *S. oleracea* was not significantly affected by As treatment (19%, on average), while the As100 treatment induced a 29% decrease in Chl_tot_ compared to the control (Table 3). The relationship between Chl_tot_ and Mg was calculated by correlation in *S. oleracea* shoots (r = 0.73, *p* ≤ 0.05).

### 2.5. Effect of Arsenic on Leaf Gas-Exchange Parameters, Chlorophyll Fluorescence, and Water Potential

The results of leaf gas-exchange parameters are presented in Table 4. All parameters, except instantaneous water-use efficiency (WUE) and intercellular CO_2_ concentration (*C*_i_), reached significantly higher values in the shoots of *S. oleracea* than *P. cretica*; however, As treatment in both plant species did not demonstrate a clear negative effect. Among the leaf gas-exchange parameters of *P. cretica*, only the net photosynthetic rate (*P*_N_) was treatment-dependent (r = –0.93, *p* ≤ 0.01); compared to the control, it significantly decreased by 4 and 9% in the As20 and As100 treatments, respectively (Table 4). In *P. cretica*, transpiration rate (*E*) was negatively affected by As treatment; however, the 23% decrease was only significant for As20 treatment. Similar to *P. cretica*, the *P*_N_ of *S. oleracea* was decreased by As100 treatment compared to the control (9%). Additionally, the stomatal conductance (*g*_s_) of *S. oleracea* decreased by 8.5 and 11% in As20 and As100 treatments, respectively (Table 4). In contrast to *P. cretica*, the *E* of *S. oleracea* was not significantly affected by As treatment compared to the control. However, the difference between As treatments was significant, and the *E* value of As100 treatment decreased by 9% compared to As20 treatment (Table 4).

**Figure 3:**
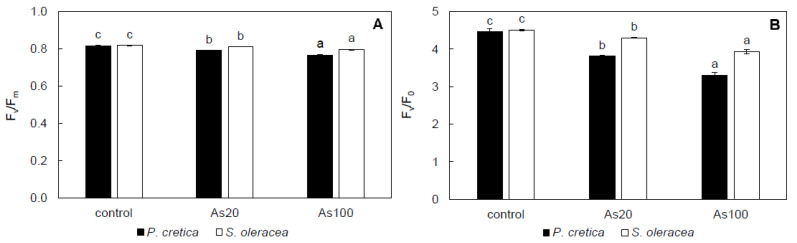
Effect of As treatments on maximal quantum yield (**A**) and maximum primary yield of PSII (**B**) in *Pteris cretica* and *Spinacia oleracea*. Values represent the mean ± SE of three biological replicates per sample. Data with the same letter are not significantly different. Different letters indicate significant differences (*p* ≤ 0.05) among treatments according to the Kruskal-Wallis test. Abbreviations: control—0 mg As/kg soil; As20—20 mg As/kg soil; As100—100 mg As/kg soil; F_v_/F_m_—maximum quantum yield of PSII; F_v_/F_0_—maximum primary yield of PSII.

The maximum quantum yield (F_v_/F_m_) and maximum primary yield of PSII (F_v_/F_0_) had a treatment-dependent response in *P. cretica* (r = –0.84 and –0.83, respectively, *p* ≤ 0.001) and *S. oleracea* shoots (r = –0.85, *p* ≤ 0.001). The *P. cretica* control was equal to the *S. oleracea* control; however, difference between plant species was significant for both As treatments. In *P. cretica* shoots, As20 and As100 treatments decreased F_v_/F_m_ by 3 and 6%, respectively, and F_v_/F_0_ by 15 and 26%, respectively (Figure 3A,B). Similar to *P. cretica*, F_v_/F_m_ and F_v_/F_0_ decreased in both As treatments in *S. oleracea*; however, the decrease was lower compared to *P. cretica*. In the As20 treatment, F_v_/F_m_ and F_v_/F_0_ of *S. oleracea* shoots decreased by 1 and 5%, respectively, compared to the control. The As100 treatment decreased F_v_/F_m_ and F_v_/F_0_ compared to the control by 3 and 13%, respectively (Figure 3A,B).

**Figure 4:**
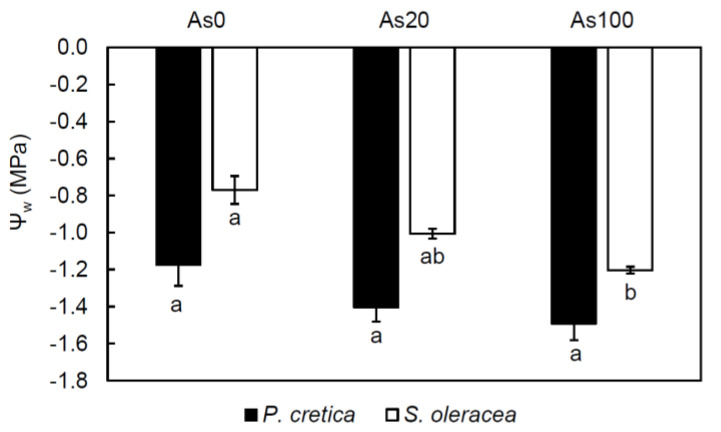
Effect of As treatments on the water potential (ψ_w_, MPa) of *Pteris cretica* and *Spinacia oleracea* in a pot experiment. Values represent the mean ± SE of three biological and two technical replicates per sample. Data with the same letter are not significantly different. Different letters indicate significant differences (*p* ≤ 0.05) among treatments according to the Kruskal-Wallis test. Abbreviations: control—0 mg As/kg soil; As20—20 mg As/kg soil; As100—100 mg As/kg soil.

A significant difference was observed in water potential (ψ_w_, Figure 4) between plants in response to As treatment; this response was treatment-dependent in *S. oleracea* shoots (r = −0.84, *p* ≤ 0.001). In *S. oleracea*, the ψ_w_ value was changed by 56% in the As100 treatment compared to the control. In contrast to *S. oleracea*, the negative effect of As treatment on ψ_w_ of *P. cretica* was not significant; however, the ψ_w_ value was changed by 27% in the As100 treatment compared to the control (Figure 4).

### 2.6. Effect of Arsenic on Free Amino Acid Metabolism

**Figure 5:**
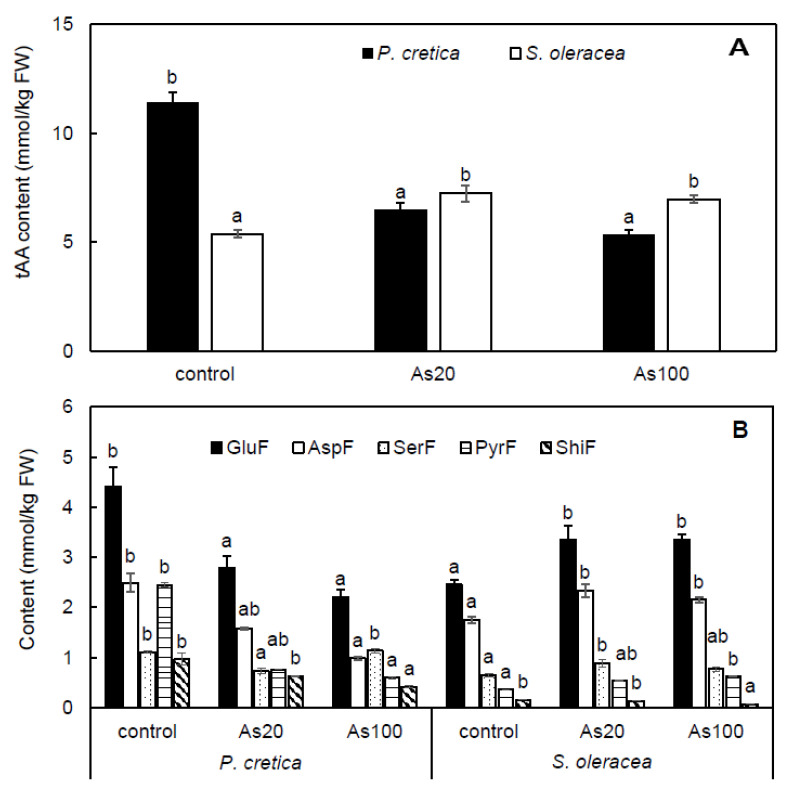
Effect of As treatments on the total content of free amino acids (**A**) and content of individual free amino acid families (**B**) in *Pteris cretica* and *Spinacia oleracea* shoots in a pot experiment. Values represent the mean ± SE of three biological and four technical replicates per sample. Data with the same letter are not significantly different. Different letters indicate significant differences (*p* ≤ 0.05) among treatments according to the Kruskal-Wallis test. Abbreviations: control—0 mg As/kg soil; As20—20 mg As/kg soil; As100—100 mg As/kg soil; tAA—total content of free amino acids; GluF—glutamate family; AspF—aspartate family; SerF—serine family; PyrF—pyruvate family; ShiF—shikimate family.

In *P. cretica* and *S. oleracea* shoots, the content of 17 free AAs and amides were measured in detectable quantities and presented as the total content of free amino acids (tAA) and content of five major AA family pathways (aspartate family—AspF, glutamate family—GluF, pyruvate family—PyrF, serine family—SerF, shikimate family—ShiF) in plant shoots (Figure 5A,B).

Significant differences between plants in response to As treatment showed the content of tAA and AA families. The tAA was treatment-dependent in *P. cretica* (r = –0.74, *p* ≤ 0.001) and *S. oleracea* shoots (r = 0.42, *p* ≤ 0.05). There was a variable response of tAA to As treatment between plant species; tAA decreased in As20 and As100 treatments of *P. cretica* by 43 and 53%, respectively, compared to the control. However, in *S. oleracea*, tAA increased by 35 and 30% in As20 and As100 treatments, respectively (Figure 5A).

In *P. cretica*, changes in family content varied among treatments; however, a clear trend in changes in family content in all treatments was shown in *S. oleracea* shoots, which decreased in the order GluF > AspF > SerF > PyrF > ShiF (Figure 5B). Among free AA families, the highest family content was determined for GluF in all treatments of both plant species (Figure 5B). The ratio of GluF ranged from 38 to 42.5% in *P. cretica* and from 46 to 48% in *S. oleracea*. The GluF values were treatment-dependent in *P. cretica* (r = –0.65, *p* ≤ 0.001) and *S. oleracea* shoots (r = 0.43, *p* ≤ 0.05). In *P. cretica* and *S. oleracea* shoots, As treatments caused the same change in GluF content as shown for tAA. Compared to the control, GluF decreased in As20 and As100 treatments of *P. cretica* by 37 and 50%, respectively, while in both As treatments, GluF in *S. oleracea* increased by 37% (Figure 5B).

**Figure 6:**
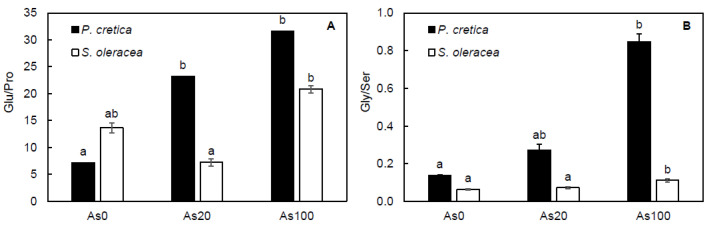
Effect of As treatments on the ratio of free glutamic acid and free proline (**A**) and ratio of free glycine and free serine (**B**) in *Pteris cretica* and *Spinacia oleracea* shoots in a pot experiment. Values represent the mean ± SE of three biological and four technical replicates per sample. Data with the same letter are not significantly different. Different letters indicate significant differences (*p* ≤ 0.05) among treatments according to the Kruskal-Wallis test. Abbreviations: control—0 mg As/kg soil; As20—20 mg As/kg soil; As100—100 mg As/kg soil; Glu—free glutamic acid; Gly—free glycine; Pro—free proline; Ser—free serine.

The main free AA from GluF was glutamic acid (Glu, Table S2), which reached 30–37% from tAA content, and decreased due to As treatment in *P. cretica* shoots, while, in *S. oleracea* shoots, it was increased by As treatments with a ratio 38.5–45% from tAA content. Control treatments of *P. cretica* and *S. oleracea* showed free proline (Pro) as the second free AA from GluF (4 and 5% from tAA, respectively), and that content was affected by As treatment (Table S2). The ratio of Glu and Pro was calculated as an important factor of Pro biosynthesis regulation from Glu, as well as an indicator of stress response in plants (Figure 6A). The Glu/Pro ratio was treatment-dependent in *P. cretica* (r = 0.76, *p* ≤ 0.001) and *S. oleracea* shoots (r = 0.73, *p* ≤ 0.001). In *P. cretica*, As treatment increased the Glu/Pro ratio, which was on average four-fold higher compared to the control, while in *S. oleracea,* a clear effect of As treatment was not determined.

In addition to the Glu/Pro ratio, the ratio of free glycine (Gly) and serine (Ser) was calculated due to the involvement of these free AAs in photosynthesis (Figure 6B). The Gly/Ser ratio was treatment-dependent in *P. cretica* (r = 0.97, *p* ≤ 0.001) and *S. oleracea* shoots (r = 0.79, *p* ≤ 0.001). However, results of SerF content were not clearly affected by As treatment in either plant species (Figure 5B). Values of free Ser showed a similar content in *P. cretica* and *S. oleracea* shoots, 10 and 11% of tAA, on average, respectively (Table S2). Compared to the control, Ser decreased due to As treatment only in *P. cretica*. In contrast, Gly content increased with As treatment in *P. cretica* (4.5% of tAA, on average) and *S. oleracea* shoots (0.8% of tAA, on average; Table S2). Compared to the control, the Gly/Ser ratio of *P. cretica* and *S. oleracea* increased in the As100 treatment (6 and 2%, respectively; Figure 6B). Both calculated ratios of selected free AAs showed significant differences between plants.

Differences between plant species were also observed in the content of free alanine (Ala) from PyrF and free phenylalanine (Phe) from ShiF (Table S2). *Pteris cretica* shoots treated with As20 and As100 accumulated lower Ala content compared to the control (by 50 and 69%, respectively); however, the decrease was only significant only for the As100 treatment (Table S2). A contrast in response to As20 and As100 treatments was determined in *S. oleracea* shoots, which accumulated higher Ala content compared to the control (97 and 194%, respectively); however, the increase was only significant for the As100 treatment (Table S2). Similarly, As100 treatment decreased Phe content in the *P. cretica* and *S. oleracea* shoots by 56 and 24%, respectively (Table S2).

## 3. Discussion

Plants show three main types of plant-soil relationships in response to increasing soil metal/metalloid concentrations [33]: (i) accumulator—metal/metalloid accumulation in shoots at both low and high soil levels; (ii) indicator—metal/metalloid uptake and transport to the shoots are regulated so that internal concentrations reflect external levels; and (iii) excluder—metal/metalloid concentrations in shoots are maintained at a low level over a wide range of soil concentrations, up to a critical soil value above which the mechanism breaks down and unrestricted transport results. Accumulators can be characterised by a shoot to root metal/metalloid content ratio of >1, whereas in excluders, the ratio is <1 [34]. Arsenic content in shoots and roots of *P. cretica* in response to As treatment showed its ability as an accumulator (Figure 1A, Table 1). Furthermore, TF values revealed high As translocation from roots to shoots, which is one of the key properties of As-hyperaccumulators, which are plants that are hypertolerant to As [35] and are able to accumulate >1000 mg As/kg biomass and have a BF >1 and TF >1 [36]. The results of As bioaccumulation and translocation confirmed *P. cretica* ‘Albo-lineata’ as an As-hyperaccumulating fern. Similar results were previously observed [21,23,28,37,38].

Different accumulation behaviours in response to As treatment in the soil revealed the As content in shoots and roots of *S. oleracea*. In roots, As bioaccumulation suggests that this plant is an excluder, while in shoots, the As content was relatively constant and low among As treatments (Figure 1B, Table 1). Furthermore, values of TF < 1 revealed the behaviour of *S. oleracea* ‘Monores’ as an excluder. *Spinacia oleracea* had an elevated metal/metalloid absorption rate [31] and, due to this behaviour, it was thought to have phytoremediation potential [30]. In contrast to our results, the As content in *S. oleracea* shoots showed that As accumulation in shoots increased with increasing As levels in growth medium or soil [30,32,39,40]. However, in these studies, the same trend was observed in roots of *S. oleracea* as was found in our *S. oleracea* plants, showing a higher As content in the roots compared to shoots, which increased with increasing As levels in growth medium or soil. In our study, the low As content in *S. oleracea* shoots could be due to their ability to restrict the entry of As into vascular bundles or As chelation and storage in the vacuoles [41].

Plants adapt to environmental changes by regulating their development and growth [1], which leads to decreased plant growth as the first visible symptom of stress [30]. Arsenic is considered phytotoxic and is expected to have adverse effects on plant growth [26] that was previously observed for the growth of *S. oleracea* [18,30,40,42] and *P. cretica* [28,37]. Similar to these findings, in our study, shoot and root dry biomass of *P. cretica* and *S. oleracea* were also affected by As concentration (Figure 2A,B). A significant decrease was observed for As100 treatment. This level was the threshold of As toxicity in non-As-hyperaccumulating plants when it was accumulated in plants’ biomass [35].

Plant growth and development are determined by the availability of nutrients, such as N, P, S, Zn, and Fe [43]. Furthermore, other nutrients, including Mn, Mg, Cu, and Ni, are important essential elements for proper physiological and metabolic functioning of plants [31,44]. In relation to As stress, a decrease in plants’ dry weight is due to the toxic As effect on various growth-related mechanisms and parameters, such as a reduction in the biosynthesis of photosynthetic pigments and a decrease in water and nutrient content [40]. In our study, the nutrient content was affected by As treatment in the shoots of both plant species (Table 2). However, the trend in changes with As treatment was different between these plants. A significant effect of As100 treatment was observed for Mg, Mn, Zn, N, and S of *P. cretica* and *S. oleracea*, while changes in Cu, Fe, and P were significant only for the lower As treatment. Despite the increase of Ni in *P. cretica* and decrease in *S. oleracea*, these changes were not significant. Similarly to our results, As induced nutrient imbalance in non-As-hyperaccumulators [45,46] as well as As-hyperaccumulators [44,47].

In this study, the same effect of As on N and S content, which increased with As100, was observed in *P. cretica* and *S. oleracea* (Table 2). Both elements are essential for plant growth, defence, development, and productivity in relation to the synthesis of AAs [43]. In contrast to our results, As toxicity regularly induced a decrease in the N content [2,30]. However, other studies also reported an increase of N by As in plants [46]. Increased N can be related to P content for coordination of the N-P balance, which is important for plant yield and energy transfer system components, e.g., ATP [43,46]. In the context of As toxicity, P content showed a difference between *P. cretica* and *S. oleracea*. A significant decrease was shown in *S. oleracea,* while in *P. cretica*, the P content was increased (Table 2). The differing P content in *P. cretica* shoots may be related to the ability of high As accumulation and translocation that can lead to higher P accumulation due to the similarity in the chemical structure of As and P. Other studies also observed inconsistent As effects on P among different plant species [25,45,46,47,48].

In addition to the important role of S, this element is necessary for plant defence, e.g., for glutathione and phytochelatins [49]. Sulfur reduced As stress in *S. oleracea* due to the enhancement of the tolerance mechanism by increasing glutathione, non-protein thiols, and phytochelatins [42]. In our study, S content increased with As100 treatment in both plant species; however, a higher content was determined in *S. oleracea* shoots, which may be related to the lower tolerance of *S. oleracea*.

The biomass of *S. oleracea* is rich in nutrients, such as Mg, Mn, P, Cu, Zn, and Fe, as well as Na, K, Ca, and I [42,50]. In our study, the content of Mg, Mn, P, Cu, Zn, and Fe was significantly higher in *S. oleracea* shoots than in *P. cretica* shoots in the control (Table 2). Differences between both plant species in response to As treatment showed a content of Mg, Mn, and Zn that was increased by As100 treatment in *P. cretica* shoots, but decreased in *S. oleracea* shoots. The same trend was observed in Fe content; however, a significant difference was observed in the lower As treatment. Iron and Mn are important for photosynthesis, e.g., Fe is chlorophyll-essential and Mn is involved in splitting water molecules necessary for photosynthesis [31]. Both increases and decreases of Fe and Mn by As treatment were observed among different plant species [37,45,46,47,48]. Antagonistic interactions between As and Fe, as well as Mn, may be due to the formation of Fe and Mn hydroxide complexes in the root system through complexation reactions and surface adsorption [46,51]. Similar changes in Fe content were observed for Cu content, which increased in *P. cretica* shoots by lower As treatment but decreased in *S. oleracea* shoots by As100 treatment (Table 2). Changes in Cu content by As treatment were observed among different plant species [37,45,46,47,48,52]. In plants, increased Cu concentration due to As stress can lead to chlorosis [45]. However, in our study, shoots of both plant species did not show any visible symptoms of As toxicity.

In plants, Zn interferes with the movement of ions across membranes, affects the activities of membrane-bound enzymes, and affects the function of permeability channels and carrier/transport proteins in the membrane [45]. Furthermore, Zn affects Fe homeostasis in plants by sensing the availability of Fe [43] and can induce chlorosis [31]. Both increases and decreases of Zn by As treatment were observed among different plant species [37,45,47,48]. In our study, Zn content increased by As100 treatment in *P. cretica* shoots, but decreased in *S. oleracea* shoots (Table 2). Zinc, together with Mn and Cu, plays an important role in metalloenzymes and metalloproteins as cofactors [53]. Decreases in these nutrients by As in *S. oleracea* shoots suggests higher activation of the As defence system compared to that in *P. cretica*.

Magnesium is the central atom in chlorophyll molecules [47]. This element is the activator of metalloenzymes and plays key roles in various plant physiological and biochemical processes [54]. Both antagonistic and synergistic interactions of As and Mg were observed among different non-As-hyperaccumulating and As-hyperaccumulating plants [25,37,41,45,46,47,48]. In our study, As affected Mg content differently between both plant species. Increase with As100 treatment was observed in *P. cretica* shoots, while Mg decreased in *S. oleracea* shoots (Table 2). In As-hyperaccumulators, elevated Mg content suggested that Mg could be used by plants to counteract As toxicity [55]. In the context of As toxicity, Mg decreases corresponded to Chl_tot_ decreases in *S. oleracea* shoots (Table 3). Absence of this relationship in *P. cretica* shoots suggests a higher As tolerance in this species.

Photosynthetic pigment content is considered vital for plant metabolism. Any harm to pigments results in severe toxicity to plants [40]. Their content is an important index for the evaluation of As tolerance in plants [56] because a decrease in pigment synthesis indicates the lack of adaptive adjustments to high As content [57]. Arsenic can affect photosynthetic pigments, the membrane system of chloroplasts, and chlorophyll fluorescence, thus reducing photosynthetic activity [58]. The As toxicity leads to a decrease in chlorophyll among different plant species [8,24,37,39,40,45,59]. Our results confirmed this effect of As on Chl_tot_ content in *S. oleracea* shoots, while in *P. cretica* shoots, the adverse As effect was not significant (Table 3). Given that a decrease in Chl_tot_ content typically takes time to manifest and is generally considered an indicator of prolonged plant stress [47], our results indicated the higher sensitivity of *S. oleracea* than *P. cretica* to As treatment. The photosynthetic pigment Car, which is important for plants due to its structural role in the organisation of photosynthetic membranes, interception of radicals, and quenching [30], was also decreased by As in non-As-hyperaccumulating plants [45,58] and As-hyperaccumulating plants [24]. However, our results did not show a significant As effect on Car content in the shoots of either plant species (Table 3). Similar results were observed for *P. cretica* [55], *S. oleraea* [30], *Ricinus communis* [52] and *Ipomoea aquatica* [60].

Among various metabolic processes, photosynthesis is one of the most significant physiological traits of plants [60]. It was reported that plants’ fitness can be affected by As due to interference with photosynthesis [56]. In our study *P*_N_, *E* and *g*_s_ in *S. oleracea* shoots were higher compared to *P. cretica* shoots (Table 4), which confirmed the lower photosynthetic capacity of ferns than seed plants [61]. Furthermore, the effect of As toxicity on photosynthesis was observed on both plant species due to changes in leaf gas-exchange parameters and chlorophyll fluorescence (Table 4, Figure 3). The reduction of photosynthesis by As stress was reported among different plant species [24,38,59,62]. In our study, *P*_N_, which directly reflects photosynthetic capacity [63], decreased with As treatment in *P. cretica* and *S. oleracea* shoots. In the context of As toxicity, *E* and *g*_s_ were affected in the shoots of both plant species (Table 4). The latter decreased with As treatment in *S. oleracea* shoots, while *P. cretica* shoots did not show a clear As effect. In both plant species, the effect of As on *E* was not clear. It was reported that *g*_s_ decreases can be caused by *E* decreases, reducing transpiration and slowing water and nutrient absorption and transportation [64]. Under As stress, both decreases and increases of these parameters were observed among different non-As-hyperaccumulating [52,64,65,66,67] and As-hyperaccumulating plants [37,55].

Chlorophyll fluorescence has emerged as a non-invasive and powerful tool to elucidate damaging modifications in the photosynthetic apparatus in stressed plants [58]. The F_v_/F_m_ and F_v_/F_0_ are important parameters frequently used to measure the maximum photochemical efficiency and activity of PS II, respectively [58]. These parameters were decreased with As treatment in *P. cretica* and *S. oleracea* shoots (Figure 3). Furthermore, the F_v_/F_m_ of As-treated plants below 0.8 confirmed the plants’ response to stress [68]. A similar effect was observed on chlorophyll fluorescence among different plant species [24,37,38,52,55,58]. A decrease in F_v_/F_m_ together with a decrease in Chl_tot_ in *S. oleracea* with As treatment can indicate a faster senescence progression [37].

In the context of As toxicity, photosynthesis can also be affected due to the influence of water uptake [8,39]. Arsenic negatively affected the ψ_w_ in plants [37,65,67]. In our study, the values of ψ_w_ showed the adverse effect of As100 treatment on *S. oleracea* shoots, while in *P. cretica* shoots, this effect was not significant (Figure 4). These results together with values of *g*_s_ suggest the higher activation of As defence in *S. oleracea* shoots, as it was reported that *g*_s_ decreases could reduce water loss in As-treated plants [8]. According to these authors, changes in the water flow dynamics may be considered an adaptive strategy to regulate metal/metalloid uptake and translocation, thus avoiding accumulation and toxicity.

Changes in AAs content with As toxicity were observed among different non-As-hyperaccumulating plant species [11,18,19], as well as among different As-hyperaccumulating plants [20,21]. The AAs play important roles in plants as sources of building blocks for membrane proteins and enzymes and act as substrates of essential primary metabolites and precursors of secondary metabolites [22,69]. Free AA accumulation serves as storage for C and N and is related to a plant’s defence system under stress [22]. Our results showed an increase of free AA by As treatment in *S. oleracea* shoots, whereas a decrease was observed in *P. cretica* shoots (Figure 5A), indicating a change of regulation of AA biosynthesis [18]. This different response to As toxicity suggests higher activation of the defence system in *S. oleracea* shoots compared to higher tolerance of *P. cretica*.

In plants, AA synthesis occurs by various metabolic pathways that can be strongly affected by different stressors [70]. The main family found in *P. cretica* and *S. oleracea* shoots was GluF, with similar AA accumulation in both plant species (Figure 5B). This family was more strongly regulated under stress [70], including As [21]. Together with AspF, these transport and storage forms of free AAs are related to C and N assimilation after disruption of the C/N ratio [38]. Results of *S. oleracea* showed increase of GluF and AspF while in the shoots of *P. cretica* these families decreased. The results of *S. oleracea* suggested higher As toxicity due to disruption of the C/N ratio. The most abundant free AA of GluF was Glu, which is an AA connected to the tricarboxylic acid (TCA) cycle, the primary source of energy for metabolism [71]. Different changes in Glu with As treatment between *P. cretica* and *S. oleracea* confirmed previously published results that the glutamine synthetase/glutamate synthase (GS/GOGAT) cycle is responsive to As [21]. In our study, the second most abundant free AA of GluF was Pro, which plays a role in the growth and stress response of plants [71]. In plants under stress, Pro is regularly accumulated [72]. However, in this study, control *P. cretica* plants had a higher Pro content than As treated plants, while Pro varied in *S. oleracea* (Table S2). These results are related to higher yield of *P. cretica* biomass that indicate use of Pro for biosynthesis of Pro/hydroxyproline-rich glycoproteins/proteins [73,74]. In the context of As toxicity, the ratio of Glu/Pro as an important factor of Pro biosynthesis regulation from Glu was calculated. Plants with a higher Glu/Pro ratio are better adapted to stress [21,75], which was also suggested by our results for *P. cretica* (Figure 6A). Furthermore, the increase of Glu/Pro in *P. cretica* indicated the activation of glutamate kinase or Δ^1^-pyrroline-5-carboxylate synthetase regulation to Pro biosynthesis from Glu [76]. However, the increase in Glu accumulation in *S. oleracea* shoots indicated the depletion of assimilated C that is necessary for AA biosynthesis [38]. Further, results attributed the Glu role to the formation of As conjugates with phytochelatins [42].

Under different stressors, AAs of SerF play a role in plants’ defence system, e.g., due to the phosphorylated pathway of Ser biosynthesis and due to biosynthesis of phytochelatins and antioxidant metabolites from Gly and cysteine [69]. As mentioned previously, in non-As-hyperaccumulating plants, As complexation with phytochelatins has a key role in As detoxification, while As-hyperaccumulating plants had less of a tendency for complex formation [13,26,27]. Arsenic hyperaccumulation in ferns is a combination of high As uptake and translocation, coupled with a high tolerance to damaging As effects by means of sequestration at the cellular level, enhanced antioxidant responses, and low reactive oxygen species concentrations [4,77]. In plant leaves, the ratio of Gly/Ser is a marker for the rate of photorespiration because these AAs are the only two directly in the photorespiratory C recycling pathway [78]. This ratio increased with net CO_2_ uptake. However, in our study, the Gly/Ser ratio increased in *P. cretica* and *S. oleracea* shoots, while *P*_N_ decreased in both plant species (Table 3, Figure 6B). These results, together with different accumulations of free Gly between plant species, showed their differences in response to As toxicity. In *S. oleracea*, the increase in Gly and Ser content suggested an increase in the photorespiration pathway for the reduction of adverse As effects in the photosystem.

Other AAs linked to plants’ response under stress are Ala and Phe [79,80]. In plants, an increase in Ala under stress suggests that glycolysis and respiration increased to sustain the higher energy demand of stressed plants or to provide C skeletons for the photorespiratory cycle [80]. Furthermore, Ala in plants has a role in intracellular pH regulation [81]. This free AA showed a difference between *P. cretica* and *S. oleracea* in response to As treatment (Table S2). The results of *S. oleracea* suggested higher As toxicity due to an increase in Ala, which is related to a disturbance in the Ala aminotransferase reaction and increased Ala synthesis [82]. Similar results were confirmed in relation to As exposure among non-As-hyperaccumulating plant species [18,19,83,84]. In the context of plants’ responses to different stressors, Phe serves as a precursor for the phenylpropanoid pathway, which produces a wide range of antioxidative metabolites and phenolic compounds [79]. Previous studies showed various changes in Phe content among different plant species under As stress [19,21]. In our study, despite the decrease of Phe with As100 treatment in both plant species, Phe content was higher in *P. cretica* shoots, which suggests a higher tolerance of this As-hyperaccumulating fern. Downregulation of Phe may directly reduce the biosynthesis of caffeic aldehyde, which plays an important role in the formation of plant lignin and can scavenge excess H_2_O_2_ [41].

## 4. Materials and Methods

### 4.1. Plant Material and Pot Experiment

A pot experiment was conducted to evaluate the effect of As treatment on two plant species with different As accumulation abilities. *Pteris cretica* L. ‘Albo-lineata’ plants were purchased at the 10-frond stage from the garden centre Tulipa Praha (Czech Republic). Seeds of spinach, *S. oleracea* L. ‘Monores’ from the Semo company were purchased from the store (Czech Republic) and were sown directly to the soil in pots.

The pot experiment was arranged as a randomised design with three biological repeats (pot) of three treatments: control (without added As), As20 (20 mg As/kg soil), and As100 (100 mg As/kg soil). Two As doses were chosen to represent low and high soil contamination and to reflect the two different levels of As toxicity and sensitivity of *P. cretica* and *S. oleracea*. The experiment was carried out under greenhouse conditions from April to June (natural photoperiod (temperature 22–24 °C during the day and 15–18 °C at night; relative humidity ~60%). Replicates of *P. cretica* contained one plant per pot, while *S. oleracea* replicates contained ten plants per pot.

Five kilograms of Haplic Chernozem from a non-polluted area in Prague-Suchdol, Czech Republic (total organic carbon 18.3 g/kg, cation-exchange capacity 258 mmol_c_/kg, pH_KCl_ 7.1, pseudo-total As 16 mg/kg, water soluble As 0.15 mg/kg, and As extraction efficiency 20%) were used per pot. Each kilogram of soil was mixed with 0.5 g N, 0.16 g P, and 0.4 g K (applied as NH_4_NO_3_ and K_2_HPO_4_). Subsequently, the soil was spiked with As (Na_2_HAsO_4_·7 H_2_O). The background soil As content was not included in the applied As dose, and the difference between the control and As treatment equalled the As spiked dose plus 20% from the total As content.

*Spinacia oleracea* plants were harvested 40 days after sowing, with shoots separated from the roots. *Pteris cretica* plants were harvested 90 days after growing in the pots, with shoots separated from the roots. Shoots were partitioned, with one portion immediately frozen in liquid nitrogen and stored at –80 °C until analysis for free AAs content determination, while the other portion was oven-dried to a constant weight (three days at 40 °C) and homogenised for elements analysis. Roots were washed with demineralised water, oven-dried to a constant weight (three days at 40 °C), and homogenised for elements analysis.

### 4.2. Analysis of Arsenic and Elements

The elements content was determined by an Agilent 720 inductively coupled plasma-optical emission spectrometer (ICP-OES; Agilent Technologies Inc., Santa Clara, CA, USA) after low-pressure microwave digestion. Homogenised dry plant material (0.5 ± 0.05 g) was digested in 10 mL of a 4:1 (*V*/*V*) mixture of HNO_3_ and H_2_O_2_ in an Ethos 1 device (MLS GmbH, Leutkirch im Allgäu, Germany). After cooling, the digested sample was diluted to 50 mL with demineralised water. A certified reference material (CRM NIST 1573a Tomato leaves) was mineralised under the same conditions for quality assurance.

### 4.3. Analysis of Total Nitrogen

Homogenised dry plant material (1.0 ± 0.05 g) was decomposed by a liquid ashing procedure in H_2_SO_4_ solution (1:20 *m*/*V*) and analysed by the Kjeldahl method using a Vapodest 50s distillation system (Gerhardt Gmbh & Co. KG., Bonn, Germany), as previously described [85].

### 4.4. Gas-Exchange Parameter Measurements

Leaf gas-exchange parameters (*P*_N_, *C*_i_, *E*, *g*_s_) were measured under photosynthetic steady-state conditions (performed between 8:00 and 11:30 Central European Time) using a portable gas exchange system LCpro+ (ADC BioScientific, Ltd., Hoddesdon, UK), as previously described [37]. Instantaneous water-use efficiency was calculated as WUE = P_N_/E.

### 4.5. Determination of Total Chlorophyll and Carotenoids

The Chl_tot_ and Car content was measured photometrically with an Evolution 2000 UV-Vis (Thermo Fisher Scientific Inc., Waltham, MA, USA), as previously described [37]. Briefly, fresh leaves (0.5 cm^2^) were incubated in the dark with 1 mL dimethylformamide. The absorbance of the extracts was measured at wavelengths of 480, 646.8, and 663.8 nm. Values at 710 nm were subtracted from these measurements. The Chl_tot_ and Car content were calculated as previously described [86,87], respectively, and normalised by the leaf area.

### 4.6. Chlorophyll Fluorescence Measurements

The minimum chlorophyll Chl *a* fluorescence (F_0_) and the maximum Chl *a* fluorescence (F_m_) were measured with a portable fluorometer OSI 1 FL (Opti-Sciences, ADC, BioScientific, Ltd., UK), as previously described [88]. Briefly, F_0_ and F_m_ were measured with 0.8 s excitation pulse (660 nm) and saturation intensity of 15,000 µmol/m^2^s after 20 min of dark adaptation in the leaves. The maximum quantum yield of PSII (F_v_/F_m_) was calculated as F_v_/F_m_ = (F_m−_F_0_)/F_m_.

### 4.7. Water Potential

The water potential was measured using a dew point PotentiaMeter (Decagon Devices, Inc., Pullman, WA, USA), as previously described [37]. Briefly, leaves were placed in a disposable syringe, frozen at −18 °C, thawed, and sap flow was pushed out into the measuring chamber of the instrument.

### 4.8. Analysis of Free Amino Acids

Free AAs were extracted and derivatised as previously described [38]. Briefly, fresh plant material (1.0 ± 0.05 g) was extracted with 15 mL of a 7:3 (*V*/*V*) mixture of methanol and twice distilled H_2_O. Extracts were derivatised using an EZ:faast kit (Phenomenex, USA). The prepared samples were analysed on a Hewlett Packard 6890N/5975 MSD gas chromatography-mass spectrometry system (GC-MS; Agilent Technologies, Santa Clara, CA, USA), as previously described [75].

### 4.9. Statistical Analyses

Statistical analyses were performed with Statistica 12.0 software (StatSoft, Inc., Tulsa, OK, USA). All data were checked for homogeneity of variance and normality (Levene and Shapiro-Wilk tests). Data did not meet the conditions for the use of analysis of variance and were thus evaluated by the nonparametric Kruskal-Wallis test. Significant differences were assessed as the effect of (i) treatment on measured parameters (description by symbols in Figures and Tables) and (ii) different species (description in text). Correlation analysis was performed using Pearson’s linear correlation (r; *p* ≤ 0.05).

## 5. Conclusions

The study of the physiological and metabolic parameters was performed on two plant species differently adapted to As uptake. Different plants’ adaptation is important to clarify the stress response of various sensitive plants to As toxicity and to evaluate the damage caused by As stress. In our study, exposure of *P. cretica* and *S. oleracea* to two As concentrations (20 and 100 mg/kg) caused changes in physiological and metabolic parameters; however, the higher concentration had a more significant effect. The fitness of plants was estimated by determining changes in growth, nutrient content, gas-exchange parameters, chlorophyll fluorescence, water potential, photosynthetic pigment content, and AA metabolism. The mechanism of As accumulation differed between both plants. *Pteris cretica* maintained the characteristics of an As-hyperaccumulating plant, such as high bioaccumulation and translocation, while *S. oleracea* exhibited very low translocation and thus higher As accumulation in the roots, suggesting that this species is an As-root excluder. Furthermore, *P. cretica* exhibited a less impaired nutrient balance, photosynthesis, and AA metabolism, which allowed better adaptation of this plant species to As toxicity. However, *S. oleracea* reflected a lower tolerance to As toxicity. In this plant species, growth inhibition was related to a decrease in photosynthesis, nutrient and photosynthetic pigment content, chlorophyll fluorescence, and water potential, as well as an increase in AA content. Compared to *P. cretica*, changes in the studied parameters in *S. oleracea* indicated higher sensitivity already at low As concentration.

## Figures and Tables

**Table 1:** Bioaccumulation factor (BF = content in the plant part/content in the soil) and translocation factor (TF = content in the shoots/content in the roots) of *Pteris cretica* and *Spinacia oleracea*. Treatment abbreviations: control—0 mg As/kg soil; As20—20 mg As/kg soil; As100—100 mg As/kg soil. Values represent the mean ± SE of three biological and two technical replicates per sample. Data with the same letter are not significantly different. Different letters indicate significant differences (*p* ≤ 0.05) among treatments according to the Kruskal-Wallis test.

	*P. cretica*			*S. oleracea*		
	TF	BF (Shoots)	BF (Roots)	TF	BF (Shoots)	BF (Roots)
control	1.82 ± 0.06 ^a^	2.65 ± 0.02 ^a^	1.46 ± 0.06 ^a^	0.67 ± 0.02 ^b^	0.13 ± 0.01 ^b^	0.20 ± 0.01 ^a^
As20	3.28 ± 0.06 ^ab^	29.34 ± 0.13 ^ab^	8.96 ± 0.11 ^b^	0.26 ± 0.01 ^ab^	0.10 ± 0.01 ^ab^	0.41 ± 0.01 ^ab^
As100	5.53 ± 0.25 ^b^	34.96 ± 0.12 ^b^	6.35 ± 0.32 ^ab^	0.05 ± 0.01 ^a^	0.05 ± 0.01 ^a^	1.10 ± 0.01 ^b^

**Table 2:** Nutrient content in *Pteris cretica* and *Spinacia oleracea* shoots. Values represent the mean ± SE of three biological and two technical replicates per sample. Data with the same letter are not significantly different. Different letters indicate significant differences (*p* ≤ 0.05) among treatments according to the Kruskal-Wallis test. Treatment abbreviations: control—0 mg As/kg soil; As20—20 mg As/kg soil; As100—100 mg As/kg soil.

	*P. cretica*			*S. oleracea*		
	Control	As20	As100	Control	As20	As100
N (g/kg DW)	26.22 ± 0.14 ^a^	28.54 ± 0.10 ^ab^	30.43 ± 0.02 ^b^	42.42 ± 0.29 ^a^	44.12 ± 0.22 ^ab^	47.19 ± 0.25 ^b^
Fe (g/kg DW)	0.12 ± 0.01 ^a^	0.22 ± 0.01 ^b^	0.19 ± 0.01 ^ab^	0.25 ± 0.01 ^b^	0.16 ± 0.01 ^a^	0.21 ± 0.01 ^ab^
Mg (g/kg DW)	3.44 ± 0.02 ^a^	3.87 ± 0.02 ^ab^	4.25 ± 0.03 ^b^	12.01 ± 0.03 ^b^	11.56 ± 0.01 ^ab^	9.06 ± 0.11 ^a^
S (g/kg DW)	1.61 ± 0.01 ^a^	2.19 ± 0.02 ^ab^	2.95 ± 0.01 ^b^	2.28 ± 0.01 ^a^	2.88 ± 0.01 ^ab^	3.46 ± 0.01 ^b^
P (g/kg DW)	2.96 ± 0.01 ^a^	4.15 ± 0.02 ^b^	3.86 ± 0.02 ^ab^	5.43 ± 0.02 ^b^	5.15 ± 0.08 ^ab^	3.6 ± 0.05 ^a^
Mn (mg/kg DW)	56.40 ± 0.40 ^a^	63.68 ± 0.69 ^ab^	65.39 ± 0.29 ^b^	145.02 ± 0.54 ^b^	108.68 ± 0.57 ^ab^	106.48 ± 0.90 ^a^
Zn (mg/kg DW)	24.38 ± 0.19 ^a^	29.00 ± 0.12 ^ab^	33.68 ± 0.14 ^b^	88.00 ± 0.55 ^b^	72.70 ± 0.05 ^ab^	48.32 ± 0.15 ^a^
Cu (mg/kg DW)	8.10 ± 0.05 ^a^	11.04 ± 0.01 ^b^	10.64 ± 0.04 ^ab^	8.91 ± 0.04 ^b^	7.99 ± 0.06 ^ab^	7.71 ± 0.09 ^a^
Ni (mg/kg DW)	0.88 ± 0.08 ^a^	0.88 ± 0.04 ^a^	1.90 ± 0.02 ^a^	0.94 ± 0.08 ^a^	0.84 ± 0.02 ^a^	0.60 ± 0.04 ^a^

**Table 3:** Effect of As treatments on photosynthetic pigments in *Pteris cretica* and *Spinacia oleracea* shoots. Values represent the mean ± SE of three biological and three technical replicates per sample. Data with the same letter are not significantly different. Different letters indicate significant differences (*p* ≤ 0.05) among treatments according to the Kruskal-Wallis test. Abbreviations: control—0 mg As/kg soil; As20—20 mg As/kg soil; As100—100 mg As/kg soil; Chl_tot_—total content of chlorophylls; Car—carotenoids.

	*P. cretica*		*S. oleracea*	
	Chl_tot_ (g/m^2^)	Car (g/m^2^)	Chl_tot_ (g/m^2^)	Car (g/m^2^)
control	0.35 ± 0.08 ^a^	0.05 ± 0.01 ^a^	0.30 ± 0.02 ^b^	0.05 ± 0.01 ^a^
As20	0.23 ± 0.06 ^a^	0.03 ± 0.01 ^a^	0.25 ± 0.01 ^ab^	0.05 ± 0.01 ^a^
As100	0.22 ± 0.04 ^a^	0.03 ± 0.01 ^a^	0.21 ± 0.03 ^a^	0.04 ± 0.01 ^a^

**Table 4:** Effect of As treatments on leaf gas exchange parameters of *Pteris cretica* and *Spinacia oleracea* in a pot experiment. Values represent the mean ± SE of three biological replicates per sample. Data with the same letter are not significantly different. Different letters indicate significant differences (*p* ≤ 0.05) among treatments according to the Kruskal-Wallis test. Abbreviations: control—0 mg As/kg soil; As20—20 mg As/kg soil; As100—100 mg As/kg soil; *P*_N_—net photosynthetic rate; *C*_i_—intercellular CO_2_ concentration; *E*—transpiration rate; *g*_s_—stomatal conductance; WUE (=*P*_N_/*E*)—water-use efficiency.

*P. cretica*	P_N_ (μmol/m^2^ s)	C_i_ [μmol(CO_2_)/mol]	E [mmol(H_2_O)/m^2^s]	g_s_ [mol(H_2_O)/m^2^s]	WUE [mol(CO_2_)/mol(H_2_O)]
control	8.08 ± 0.03 ^c^	298.60 ± 12.15 ^a^	0.78 ± 0.02 ^b^	0.03 ± 0.001 ^b^	10.39 ± 0.21 ^ab^
As20	7.72 ± 0.01 ^b^	319.60 ± 16.59 ^a^	0.60 ± 0.03 ^a^	0.02 ± 0.002 ^a^	13.45 ± 0.76 ^b^
As100	7.31 ± 0.01 ^a^	302.13 ± 9.51 ^a^	0.77 ± 0.01 ^b^	0.03 ± 0.001 ^b^	9.48 ± 0.17 ^a^
S. oleracea					
control	11.13 ± 0.26 ^b^	297.25 ± 4.53 ^b^	2.65 ± 0.03 ^ab^	0.20 ± 0.004 ^b^	4.20 ± 0.09 ^b^
As20	10.73 ± 0.14 ^b^	273.39 ± 3.63 ^a^	2.78 ± 0.03 ^b^	0.18 ± 0.004 ^a^	3.87 ± 0.07 ^a^
As100	10.14 ± 0.18 ^a^	279.32 ± 1.07 ^ab^	2.54 ± 0.04 ^a^	0.18 ± 0.005 ^a^	4.01 ± 0.06 ^ab^

## Data Availability

The data presented in this study are available in article and supplementary material.

## Supplementary Materials

The following are available online at https://www.mdpi.com/article/10.3390/plants10102009/s1, Table S1: Pearson’s linear correlation (r) between As treatments and nutrient content in *Pteris cretica* and *Spinacia oleracea* shoots, Table S2: Effect of As treatment on selected free amino acid content in *Pteris cretica* and *Spinacia oleracea* shoots. Values represent the mean ± SE of three biological and four technical replicates per sample. Data with the same letter are not significantly different. Different letters indicate significant differences (*p* ≤ 0.05) among treatments according to the Kruskal-Wallis test.
