# Supplementary material for: Arsenic Toxicity-Induced Physiological and Metabolic Changes in the Shoots of Pteris cretica and Spinacia oleracea
Source: Plants (Basel). 2021 Sep 25;10(10):2009. doi: 10.3390/plants10102009 (PMC8540401; doi:10.3390/plants10102009)
Supplement: Supplementary file 1 [file plants-10-02009-s001.zip › Table S1.pdf]

Table S1. Pearson's linear correlation (r) between As treatments and nutrient content in *Pteris cretica* and *Spinacia oleracea* shoots. n = 9 each treatment. * $p \leq 0.05$, ** $p \leq 0.01$, *** $p \leq 0.001$, NS — not significant.

**As treatment**	***P. cretica* - r**	***S. oleracea* - r**
**× N**	0.92 ***	0.97 ***
**× Fe**	NS	NS
**× Cu**	NS	−0.79 *
**× Ni**	0.97 ***	−0.88 **
**× Mn**	0.77 *	−0.69 *
**× Mg**	0.93 ***	−0.99 ***
**× Zn**	0.95 ***	−0.98 ***
**× S**	0.97 ***	0.94 ***
**× P**	NS	−0.99 ***
