# Supplementary material for: Arsenic Toxicity-Induced Physiological and Metabolic Changes in the Shoots of Pteris cretica and Spinacia oleracea
Source: Plants (Basel). 2021 Sep 25;10(10):2009. doi: 10.3390/plants10102009 (PMC8540401; doi:10.3390/plants10102009)
Supplement: Supplementary file 1 [file plants-10-02009-s001.zip › Table S2.pdf]

Table S2. Effect of As treatment on selected free amino acid content in *Pteris cretica* and *Spinacia oleracea* shoots. Values represent mean ± SE of three biological and four technical replicates per sample. Data with the same letter are not significantly different. Different letters indicate significant differences ($p \leq 0.05$) among treatments according to the Kruskal-Wallis test. Abbreviations: control — 0 mg As/kg soil; As20 — 20 mg As/kg soil; As100 — 100 mg As/kg soil; Ala — free alanine; Glu — free glutamic acid; Pro — free proline; Gly — free glycine; Phe — free phenylalanine; Ser — free serine.

**Amino acid**	***P. cretica***			***S. oleracea***		
	**control**	**As20**	**As100**	**control**	**As20**	**As100**
**Ala (μmol/kg FW)**	633.17± 12.20[b]	315.46± 4.46[ab]	196.09± 3.94[a]	145.95± 6.09[a]	287.92± 10.22[ab]	429.36± 6.27[b]
**Glu (μmol/kg FW)**	3448.24± 387.57[b]	2443.81± 245.28[ab]	1981.28± 158.82[a]	2160.81± 108.05[a]	2839.12± 278.71[ab]	3126.21± 109.71[b]
**Pro (μmol/kg FW)**	482.88± 9.78[b]	105.51± 1.52[ab]	62.79± 1.25[a]	160.09± 4.64[a]	390.02± 9.43[b]	149.94± 1.23[a]
**Gly (μmol/kg FW)**	132.77± 2.87[a]	146.86± 2.12[a]	514.79± 10.03[b]	38.42± 1.45[a]	55.95± 1.43[ab]	75.41± 2.50[b]
**Phe (μmol/kg FW)**	957.24± 123.99[b]	599.42± 16.38[b]	417.97± 20.96[a]	80.90± 3.73[b]	84.25± 2.60[b]	61.76± 1.13[a]
**Ser (μmol/kg FW)**	972.34 ± 26.48[b]	582.44± 59.62[a]	618.67± 34.02[a]	612.42 ± 27.11[a]	829.68 ± 81.35[a]	698.39± 42.62[a]
